# Supplementary material for: Characterisation of bacteria isolated from the stingless bee, Heterotrigona itama, honey, bee bread and propolis
Source: PeerJ. 2019 Aug 22;7:e7478. doi: 10.7717/peerj.7478 (PMC6708576; doi:10.7717/peerj.7478)
Supplement: Supplemental Information 1 — The results of TPC was obtained from samples grown on nutrient agar and incubated at 37 °C. [file peerj-07-7478-s001.docx]

**Table S1. Number of total plate count (TPC) obtained from *H. itama* nest products**. The results of TPC was obtained from samples grown on nutrient agar and incubated at 37 °C.

| Sampling location | Total plate count (cfu/g) ± SD | | |
| --- | --- | --- | --- |
|  | Propolis | Honey | Bee bread |
| Yayasan Al-Jenderami | 1.8 × 10^4^ ± 5.8 × 10^4^ | 8.0 × 10^3^ ± 1.0 × 10^3^ | 8.6 × 10^3^ ± 5.8 × 10^3^ |
| Ladang nangka PASFA | 9.0 × 10^3^ ± 2.6 × 10^2^ | ND | 5.0 × 10^2^ ± 2.6 × 10^2^ |
| Giant B Farm | 9.0 × 10^3^ ± 2.0 × 10^3^ | 6.3 × 10^2^ ± 3.2 × 10^2^ | 1.1 × 10^3^ ± 5.8 × 10^3^ |
| Ladang 10 UPM | 6.3 × 10^3^ ± 5.5 × 10^2^ | 7.0 × 10^2^ ± 4.4 × 10^2^ | ND |
| ND = No detectable growth. The one-way ANOVA test showed significant differences (p=0.041) between honey, bee bread and propolis from different locations. | | | |
