# Supplementary material for: Characterisation of bacteria isolated from the stingless bee, Heterotrigona itama, honey, bee bread and propolis
Source: PeerJ. 2019 Aug 22;7:e7478. doi: 10.7717/peerj.7478 (PMC6708576; doi:10.7717/peerj.7478)
Supplement: Supplemental Information 2 — Bacterial isolates were grown at 37 °C on nutrient agar for macroscopic and microscopic analysis. [file peerj-07-7478-s002.docx]

**Table S2. Morphological and biochemical characterisation of bacterial isolates**. Bacterial isolates were grown at 37 °C on nutrient agar for macroscopic and microscopic analysis.

| Origin of isolation | Isolate | Morphological and biochemical characterization | | | | | | | | | | | | | | |
| --- | --- | --- | --- | --- | --- | --- | --- | --- | --- | --- | --- | --- | --- | --- | --- | --- |
|  |  | Size | Form | Elevation | Margin | Opacity | Texture | Color | Gram-stain | Shape | Endospore | Catalase | Amylolytic | Proteolytic | Lipolytic | Cellulolytic |
| Propolis | PD1^a^ | M | Irregular | Flat | Entire | Opaque | Moist | White | + | Rod-shaped | + | + | + | + | - | + |
|  | PD3^a^ | S | Circular | Convex | Entire | Translucent | Moist | White | + | Rod-shaped | + | + | - | - | - | - |
|  | PD4^a^ | S | Circular | Convex | Entire | Opaque | Moist | White | + | Rod-shaped | + | + | - | - | - | - |
|  | PD5^a^ | M | Circular | Flat | Entire | Opaque | Moist | White | + | Rod-shaped | + | + | - | + | + | + |
|  | PD6^a^ | M | Circular | Flat | Entire | Opaque | Moist | White | + | Rod-shaped | + | + | - | + | + | + |
|  | PD7^a^ | M | Irregular | Flat | Entire | Opaque | Moist | White | + | Rod-shaped | + | + | + | + | - | + |
|  | PD9^a^ | L | Umbonate | Convex | Entire | Opaque | Viscid | White | + | Rod-shaped | + | + | + | + | + | + |
|  | PD10^a^ | M | Irregular | Flat | Entire | Opaque | Moist | White | + | Rod-shaped | + | + | + | + | - | + |
|  | *PD12^a^ | S | Circular | Convex | Entire | Translucent | Moist | Yellowish | - | Rod-shaped | - | + | - | - | - | + |
|  | PD13^a^ | M | Irregular | Raised | Entire | Opaque | Moist | White | + | Rod-shaped | + | + | + | + | - | + |
|  | PD14^a^ | S | Circular | Convex | Entire | Translucent | Moist | White | + | Rod-shaped | + | + | - | - | - | - |
|  | PD16^a^ | M | Irregular | Flat | Entire | Opaque | Moist | White | + | Rod-shaped | + | + | + | + | - | + |
|  | *PG1^b^ | S | Circular | Convex | Entire | Translucent | Moist | Yellowish | - | Rod-shaped | - | + | - | - | - | + |
|  | PM1^c^ | M | Circular | Raised | Entire | Opaque | Moist | White | + | Rod-shaped | + | + | + | + | - | + |
|  | PM2^c^ | M | Circular | Raised | Entire | Opaque | Moist | White | + | Rod-shaped | + | + | + | + | - | + |
|  | PM3^c^ | M | Circular | Raised | Entire | Opaque | Moist | White | + | Rod-shaped | + | + | + | + | - | + |
|  | *PM4^c^ | S | Circular | Convex | Entire | Translucent | Moist | Yellowish | - | Rod-shaped | - | + | - | + | - | + |
|  | PU1^d^ | L | Umbonate | Convex | Entire | Opaque | Viscid | White | + | Rod-shaped | + | + | + | + | + | + |
| Bee bread | BD1^a^ | M | Irregular | Flat | Entire | Opaque | Moist | White | + | Rod-shaped | + | + | + | + | - | + |
|  | BD2^a^ | M | Irregular | Flat | Entire | Opaque | Moist | White | + | Rod-shaped | + | + | + | + | - | + |
|  | BD3^a^ | M | Irregular | Flat | Entire | Opaque | Dry | White | + | Rod-shaped | + | + | - | + | + | + |
|  | BD4^a^ | S | Circular | Convex | Entire | Opaque | Moist | Whitish-yellow | + | Rod-shaped | + | + | - | - | + | + |
|  | BD5^a^ | M | Irregular | Flat | Entire | Opaque | Moist | White | + | Rod-shaped | + | + | + | + | - | + |
|  | BD6^a^ | M | Irregular | Flat | Entire | Opaque | Moist | White | + | Rod-shaped | + | + | + | + | - | + |
|  | BD7^a^ | M | Irregular | Flat | Entire | Opaque | Moist | White | + | Rod-shaped | + | + | + | + | - | + |
|  | BD8^a^ | M | Circular | Raised | Entire | Opaque | Moist | White | + | Rod-shaped | + | + | + | + | - | - |
|  | BD9^a^ | M | Circular | Flat | Entire | Opaque | Moist | White | + | Rod-shaped | + | + | - | + | + | + |
|  | *BG1^b^ | S | Irregular | Raised | Entire | Opaque | Dry | Yellowish | + | Filamentous | + | + | - | - | - | + |
|  | BM1^c^ | M | Circular | Raised | Entire | Opaque | Moist | White | + | Rod-shaped | + | + | + | + | - | + |
|  | BM2^c^ | M | Irregular | Flat | Entire | Opaque | Moist | White | + | Rod-shaped | + | + | + | + | - | + |
|  | BM3^c^ | M | Irregular | Flat | Entire | Opaque | Moist | White | + | Rod-shaped | + | + | + | + | - | + |
|  | BM4^c^ | S | Circular | Convex | Entire | Opaque | Moist | Whitish-yellow | + | Rod-shaped | + | + | - | + | - | + |
| Honey | HD1^a^ | M | Irregular | Flat | Entire | Opaque | Moist | White | + | Rod-shaped | + | + | + | + | - | + |
|  | HD2^a^ | M | Irregular | Flat | Entire | Opaque | Moist | White | + | Rod-shaped | + | + | + | + | - | + |
|  | HD3^a^ | M | Irregular | Flat | Entire | Opaque | Moist | White | + | Rod-shaped | + | + | + | + | - | + |
|  | HD4^a^ | M | Irregular | Flat | Entire | Opaque | Moist | White | + | Rod-shaped | + | + | + | + | - | + |
|  | HD7^a^ | M | Irregular | Flat | Entire | Opaque | Moist | White | + | Rod-shaped | + | + | + | + | - | + |
|  | HM1^c^ | M | Irregular | Flat | Entire | Opaque | Moist | White | + | Rod-shaped | + | + | + | + | - | + |
|  | HM2^c^ | M | Irregular | Flat | Entire | Opaque | Dry | White | + | Rod-shaped | + | + | + | + | - | - |
|  | HU1^d^ | M | Irregular | Flat | Entire | Opaque | Moist | White | + | Rod-shaped | + | + | + | + | - | + |
|  | HU2^d^ | M | Irregular | Flat | Entire | Opaque | Moist | White | + | Rod-shaped | + | + | + | + | - | + |
| ^a^ Yayasan Al-Jenderami  ^b^ Ladang nangka PASFA  ^c^ Giant B Farm  ^d^ Ladang 10 UPM  *Not screened as *Bacillus* spp.  S : small (2-3 mm), M : medium (4-5 mm), L : large (> 5 mm), + : positive, - : negative | | | | | | | | | | | | | | | | |
