# Supplementary material for: Characterisation of bacteria isolated from the stingless bee, Heterotrigona itama, honey, bee bread and propolis
Source: PeerJ. 2019 Aug 22;7:e7478. doi: 10.7717/peerj.7478 (PMC6708576; doi:10.7717/peerj.7478)
Supplement: Supplemental Information 3 [file peerj-07-7478-s003.docx]

**Table S3. The comparison of 16S rRNA gene sequence of bacterial isolates from *H. itama* nest products with the 16S rRNA gene sequence in GenBank**.

| Isolates | Sequence | | | | |
| --- | --- | --- | --- | --- | --- |
|  | GenBank accession no.^a^ | Number of nucleotides^b^ | Closest phylogenetic relative^c^ | Score (gaps)^d^ | Identity |
|  |  |  |  |  | (%)^e^ |
| PD1 | KY773584 | 1391 | *Bacillus cereus* ATCC 14579 | 2523 (3) | 99.71 |
| PD7 | KY773589 | 1372 | *Bacillus cereus* ATCC 14579 | 2527 (0) | 99.93 |
| PD10 | KY777580 | 1391 | *Bacillus cereus* ATCC 14579 | 2560 (0) | 99.93 |
| PD16 | KY773593 | 1399 | *Bacillus cereus* ATCC 14579 | 2507 (1) | 99.78 |
| BD1 | KY773594 | 1431 | *Bacillus cereus* ATCC 14579 | 2381 (0) | 99.24 |
| BD2 | KY773595 | 1431 | *Bacillus cereus* ATCC 14579 | 2617 (2) | 99.72 |
| BD5 | KY777581 | 1432 | *Bacillus cereus* ATCC 14579 | 2542 (0) | 99.78 |
| BD6 | KY773598 | 1431 | *Bacillus cereus* ATCC 14579 | 2632 (1) | 99.79 |
| BD7 | KY773599 | 1431 | *Bacillus cereus* ATCC 14579 | 2442 (3) | 99.48 |
| BM2 | KY773607 | 1434 | *Bacillus cereus* ATCC 14579 | 2621 (2) | 99.72 |
| BM3 | KY773608 | 1433 | *Bacillus cereus* ATCC 14579 | 2627 (2) | 99.79 |
| HD1 | KY773580 | 1432 | *Bacillus cereus* ATCC 14579 | 2636 (0) | 99.93 |
| HD2 | KY773581 | 1424 | *Bacillus cereus* ATCC 14579 | 2625 (1) | 99.93 |
| HD3 | KY773582 | 1177 | *Bacillus cereus* ATCC 14579 | 2130 (0) | 99.66 |
| HD4 | KY777579 | 1440 | *Bacillus cereus* ATCC 14579 | 2641 (0) | 100.00 |
| HD7 | KY773583 | 1408 | *Bacillus cereus* ATCC 14579 | 2344 (1) | 98.08 |
| HM1 | KY773604 | 1396 | *Bacillus cereus* ATCC 14579 | 2540 (4) | 99.57 |
| HU1 | MG738302 | 1419 | *Bacillus cereus* ATCC 14579 | 2606 (2) | 99.79 |
| HU2 | MG738303 | 1419 | *Bacillus cereus* ATCC 14579 | 2606 (2) | 99.79 |
| PM1 | KY773610 | 1425 | *Bacillus aryabhattai* B8W22 | 2621 (0) | 99.86 |
| PM2 | KY773611 | 1375 | *Bacillus aryabhattai* B8W22 | 2529 (0) | 99.93 |
| PM3 | KY773612 | 1428 | *Bacillus aryabhattai* B8W22 | 2603 (3) | 99.58 |
| BD8 | KY773600 | 1408 | *Bacillus aryabhattai* B8W22 | 2514 (1) | 98.94 |
| BM1 | KY773606 | 1391 | *Bacillus aryabhattai* B8W22 | 2556 (2) | 99.86 |
| PD3 | KY773585 | 1433 | *Bacillus oleronius* ATCC 700005 | 2623 (0) | 99.72 |
| PD14 | KY773590 | 1432 | *Bacillus oleronius* ATCC 700005 | 2615 (1) | 99.65 |
| PD5 | KY773587 | 1353 | *Bacillus stratosphericus* 41KF2a | 2477 (1) | 99.85 |
| PD6 | KY773588 | 1379 | *Bacillus stratosphericus* 41KF2a | 2505 (0) | 99.71 |
| BD4 | KY773597 | 1428 | *Bacillus altitudinis* 41KF2b | 2628 (1) | 99.93 |
| BM4 | KY773609 | 1429 | *Bacillus altitudinis* 41KF2b | 2625 (2) | 99.86 |
| PD9 | KY773579 | 1556 | *Bacillus amyloliquefaciens* FZB42 | 2730 (4) | 99.34 |
| PU1 | MG738304 | 1415 | *Bacillus amyloliquefaciens* FZB42 | 2591 (1) | 99.72 |
| PD4 | KY773586 | 1394 | *Bacillus nealsonii* DSM 15077 | 2420 (5) | 98.47 |
| PD13 | KY773592 | 1394 | *Bacillus toyonensis* BCT-7112 | 2464 (0) | 99.78 |
| BD3 | KY773596 | 1428 | *Bacillus subtilis* JCM 1465 | 2632 (1) | 99.93 |
| BD9 | KY773601 | 1429 | *Bacillus safensis* NBRC 100820 | 2630 (1) | 99.93 |
| HM2 | KY773605 | 1379 | *Bacillus pseudomycoides* NBRC 101232 | 2471 (2) | 99.06 |
| PD12 | KY773591 | 1377 | *Enterobacter asburiae* JCM 6051 | 2375 (1) | 99.24 |
| PM4 | KY773613 | 1374 | *Enterobacter cloacae* ATCC 23373 | 2479 (6) | 99.27 |
| PG1 | KY773602 | 1358 | *Pantoea dispersa* LMG 2603 | 2289 (1) | 99.44 |
| BG1 | KY773603 | 1404 | *Streptomyces kunmingensis* NBRC 14463 | 2429 (5) | 98.62 |
| ^a^ Accession number of each sequence was provided from GenBank database. ^b^ The number of 16S rRNA gene nucleotides from the combination of 8F and 1492R primers. ^c^ Closely related species from GenBank database. ^d^ The matching score with the closest phylogenetic relative has 0.0 E value and the number of gaps in bracket. ^e^ The percentage identity with the closest phylogenetic relative of bacteria. | | | | | |
